# Common and Rare 5′UTR Variants Altering Upstream Open Reading Frames in Cardiovascular Genomics

**DOI:** 10.3389/fcvm.2022.841032

**Published:** 2022-03-21

**Authors:** Omar Soukarieh, Caroline Meguerditchian, Carole Proust, Dylan Aïssi, Mélanie Eyries, Aurélie Goyenvalle, David-Alexandre Trégouët

**Affiliations:** ^1^INSERM, Bordeaux Population Health, U1219, Molecular Epidemiology of Vascular and Brain Disorders, University of Bordeaux, Bordeaux, France; ^2^Department of Genetics, Pitié-Salpêtrière Hospital, Assistance Publique-Hôpitaux de Paris, Sorbonne Université, Paris, France; ^3^UVSQ, Inserm, END-ICAP, Université Paris-Saclay, Versailles, France

**Keywords:** open reading frame (ORF), genome wide association analysis (GWAS), Mendelian disease, non-coding mutations, polymorphism

## Abstract

High-throughput sequencing (HTS) technologies are revolutionizing the research and molecular diagnosis landscape by allowing the exploration of millions of nucleotide sequences at an unprecedented scale. These technologies are of particular interest in the identification of genetic variations contributing to the risk of rare (Mendelian) and common (multifactorial) human diseases. So far, they have led to numerous successes in identifying rare disease-causing mutations in coding regions, but few in non-coding regions that include introns, untranslated (UTR), and intergenic regions. One class of neglected non-coding variations is that of 5′UTR variants that alter upstream open reading frames (upORFs) of the coding sequence (CDS) of a natural protein coding transcript. Following a brief summary of the molecular bases of the origin and functions of upORFs, we will first review known 5′UTR variations altering upORFs and causing rare cardiovascular disorders (CVDs). We will then investigate whether upORF-affecting single nucleotide polymorphisms could be good candidates for explaining association signals detected in the context of genome-wide association studies for common complex CVDs.

## Introduction

**FIGURE 1:**
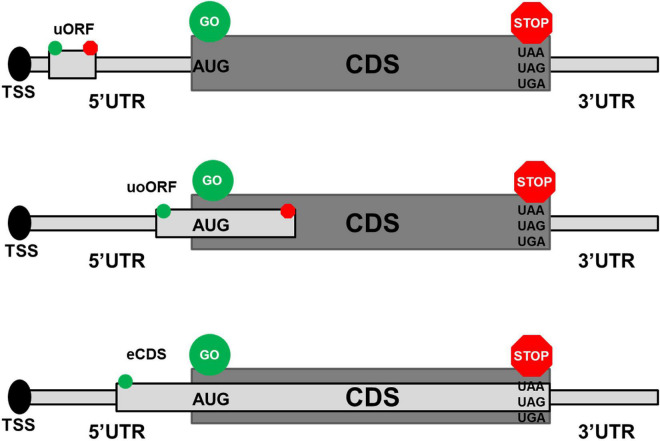
Different types of upstream open reading frames (upORFs) located in the 5′UTR of coding transcripts. The upper, middle, and lower panels show the position on coding transcripts of fully upstream ORF (uORF), overlapping ORF (uoORF), and elongated coding sequence (eCDS), respectively. The start and stop codons associated to the described upORF are indicated by green and red circles, respectively. AUG corresponds to the canonical start codon and UAA, UAG, and UGA correspond to the stop codons. TSS, transcription start site; UTR, untranslated region; CDS, coding sequence.

Upstream open reading frames (upORFs) are key regulatory elements located in the 5′untranslated (UTR) region of coding transcripts. UpORFs result from the presence of an upstream translation initiation site (uTIS) located within the 5′UTR and associated with an in-frame stop codon (uStop) located within the 5′UTR or the coding sequence (CDS). Different types of upORFs can be distinguished according to the position of the uStop with respect to the CDS (Figure 1). More precisely, when the uStop (i) is located within the 5′UTR, this results in a fully upstream ORF (uORF), (ii) is located within the CDS and is distinct from the main stop codon of the CDS, the uTIS is at the origin of an overlapping uORF (uoORF), and (iii) is the main stop codon of the CDS, this leads to an elongated CDS (eCDS). Approximately, half of the human transcripts naturally contain upORFs in their 5′UTR (1, 2) and these upORFs can contribute to modulate the production of the main protein encoded by the CDS by disturbing the translation initiation step and then the recognition of the main TIS by the ribosomes (3–5). The functional effect of a given upORF is highly variable and could be influenced by elements including the number of upORFs in the 5′UTR, their length, and the nucleotide context of the upORF as extensively discussed previously (6).

Often, the presence of upORF in general, and uoORF in particular, leads to a decrease of the expression of the main transcript (1). That could happen *via* the alteration of the translation mechanism (i.e., ribosome dissociation and ribosome stalling) or *via* transcript degradation by the non-sense-mediated decay process that recognizes the uStop as a premature stop codon (1, 7, 8). Nevertheless, under some conditions (i.e., hypoxia or cell stress), the presence of upORFs in a given transcript could be associated with an increase of the translation efficiency (9, 10). Indeed, upORF can modulate the activity of coexisting internal ribosome entry site (IRES) located on the same 5′UTR (11), thus regulating the IRES-dependent translation initiation in a context dependent manner. For instance, Chen et al., showed that the increase of fibroblst growth factor 9 (FGF9) protein levels under hypoxia happens *via* an IRES-dependent translation, regulated by the presence of a small upORF upstream to the IRES (12). In normal conditions (i.e., normaxia), FGF9 is present in low levels in human cells, thanks to the upORF-mediated translation inhibition of the CDS. Under hypoxia conditions, ribosomes probably switch from the upORF to IRES, thus activating the IRES-dependent translation and leading to efficient translation of FGF9 (12). That explains the increase of FGF9 under hypoxia conditions in cancer cells. In addition, upORFs could be translated into small-encoded peptides (SEPs) and play a regulatory role in health and disease contexts (13, 14).

High-throughput genomic studies have identified an increasingly number of single nucleotide variations (SNVs) located in 5′UTR and possibly altering upORFs by creating new ones or deleting/modifying existing ones suggesting that this kind of variants has been underestimated (15). Many of these variants have been characterized as disease causing by creating upORF and, thus, altering the production of the canonical protein, but surprisingly this has still not been investigated systematically. In fact, among the ∼4,000 disease-associated 5′UTR variants reported in different databases, the most deleterious ones are those creating or deleting uTIS or uStop, responsible of the creation or the disruption of upORFs (15). Whiffin et al. have recently shown that, among all the SNVs reported in the genome aggregation (GnomAD) database to locate in 5′UTR of 18,593 canonical transcripts, on an average of 30 SNVs per gene are variations creating a uAUG canonical initiation codon (15). They also showed that only 39 uAUG-creating and four stop-removing extremely rare variants were reported in Human Gene Mutation Database (HGMD) or likely pathogenic in ClinVar (15). Very interestingly, among these rare variants, nine uAUG-creating variants are located in genes implicated in cystic fibrosis, familial hypercholesterolemia, and hematologic diseases (16–22). Moreover, recent studies have also shown that upORF could be initiated by non-AUG codons and be disease causing (23, 24). Given the diversity of the functional implication of existing upORFs in the regulation of protein expression, the possible functional impacts of upORF-altering variants, hereafter called upSNVs, on protein expression could be highly variable. Up to very recently (25, 26), this type of genetic variants was not easily predicted by available bioinformatics tools. In addition, their functional characterization requires dedicated experimental strategies that have not yet been harmonized in order to demonstrate how they could affect gene expression and how the resulting dysregulations could lead to disease. Nevertheless, a first step in the assessment of the effect of upSNVs on the protein levels can be obtained using *in vitro* functional assays in which the 5′UTR and CDS of a given transcript are cloned in expression vectors followed by the expression of the produced vectors in human cells, both in the wild-type and upSNV contexts (27). upSNV-associated protein levels could then be evaluated by Western blot in comparison to the wild-type construct. Luciferase assays have also been widely used to study upSNVs. These assays are based on the cloning of the entire promoter of a given transcript before the coding sequence of a luciferase and the evaluation of the promoter activity in wild-type and mutant contexts *in vitro* by measuring the obtained luciferase luminescence normalized to a control vector. Additional methods used to characterize small ORFs and their potential translation into SEPs has been recently reviewed in (28). Altogether, upSNVs are still a neglected class of non-coding variations, and are often called as Variants of Unknown Significance when they are identified in routine clinical diagnosis, contributing then to medical wandering. In this work, with the aim of putting new light on upSNVs, we first provide a general overview of such type of variants known to cause rare cardiovascular disorders (CVDs). Then, we explore their potential role as candidates for explaining association signals detected in the context of genome-wide association studies (GWASs) for common complex CVDs.

## Methods

Two complementary strategies were adopted to identify rare uAUG-creating variants in CVD genes. First, we selected variants from Supplementary Table 2 of (15) reporting upSNVs from ClinVar and HGMD. Then, we looked for additional variants in ClinVar and HGMD that were not reported in Whiffin et al., and scanned research articles in PubMed using the following keywords: “upstream ORF” and “cardio-vascular.”

To investigate whether some association signals detected in GWAS for CVDs could be explained by upSNVs, we deployed MORFEE^1^ on the 1,000 Genome reference dataset (phase 3-v20130502) in order to identify all the common (allele frequency > 1%) predicted upSNVs in 5′UTR regions. In a second step, we checked whether these predicted upSNVs could be in linkage disequilibrium (LD) with lead SNVs identified in GWAS studies for coronary artery disease (CAD), stroke, venous thrombosis (VT), platelets, and lipid traits. LD information was retrieved from the European populations genetic database available through the LDlink web-based tool^2^ and from which we considered two SNVs to be in LD when the absolute value of their pairwise D’ was greater than 0.7. For CAD, GWAS loci and lead SNVs were selected from Matsui et al. (29) and Hartiala et al. (30), while Malik et al. (31) and Lindström et al. (32) were used to identify GWAS loci and corresponding lead SNVs for stroke and VT, respectively. For platelets and lipids traits, we selected all the SNVs reported in the Geospatial Resource for Agriculture Species and Pests (GRASP) server^3^ (33) as of September 2021 to associate at *p* < 5.10^–8^ with any of their related quantitative traits, including mean platelet volume, platelet count, platelet aggregation or platelets’ response to medication for platelet therapy, and high-density lipoprotein (HDL)-/low-density lipoprotein (LDL)/total cholesterol, triglycerides for lipids. Finally, this selection strategy led to a list of 749 CVD traits associated loci scrutinized for harboring common upSNVs.

## Results

### Rare upSNVs Causing Cardiovascular Disorders

This section describes in detail upSNVs known to cause rare CVDs, most of which have been cataloged in (15). Information is summarized in Table 1.

**TABLE 1:** Rare upSNVs in CVD-related diseases.

Gene (orientation)	cDNA position	Predicted effect	Disease	Databases	Classification (ClinVar)	References
*HBB* (−1)	NM_000518.5 c.-29G>A	uoORF (42 nts)	β-Thalassaemia	ClinVar	Pathogenic	1, 16
*CFTR* (1)	NM_000492.3 c.-34C>T	uoORF (108 nts)	Disseminated bronchiectasis	HGMD, ClinVar	Conflicting interpretations of pathogenicity	19
*ENG* (−1)	NM_001114753.3 c.-142A>T	uoORF (270 nts)	Hereditary Haemorrhagic TelangiectasiaT	NA	NA	77
*ENG* (−1)	NM_001114753.3 c.-127C>T	uoORF (255 nts)		HGMD	Pathogenic/Likely pathogenic	18, 35, 77
*ENG* (−1)	NM_001114753.3 c.-10C>T	uoORF (138 nts)		HGMD	Likely pathogenic	34
*ENG* (−1)	NM_001114753.3 c.-9G>A	eCDS (+ 3 nts)		HGMD	Conflicting interpretations	35
*ENG* (−1)	NM_001114753.3 c.-79C>T	uoORF (207 nts)	NA*	ClinVar	Uncertain significance	NA
*PROS1* (−1)	NM_000313.4 c.-39C>T	uoORF (156 nts)	Protein S deficiency	NA	NA	27
*F8* (−1)	NM_000132.4 c.-5A>G	uoORF (63 nts)	Hemophilia A	HGMD	NA	20
*HAMP* (1)	NM_021175.4 c.-25G>A	uAUG**	Juvenile Hereditary Hemochromatosis	HGMD	NA	15, 17
*LDLR* (1)	NM_000527.5 c.-22delC	uoORF (174 nts)	Familial Hypercholesterolaemia	ClinVar	Uncertain significance	22, 41

*uoORF, upstream overlapping Open Reading Frame; eCDS, elongated coding sequence; nts, nucleotides; NA, non-available.*

*This variant is reported in ClinVar without any clinical annotation (https://www.ncbi.nlm.nih.gov/clinvar/variation/618621/?new_evidence=false).

**No in frame stop codon predicted.

*HBB* c.-29G>A appeared to be one of the first examples of uAUG-creating variants associated with tan inherited blood disorder, β-thalassemia characterized by marked reduce or absence of the beta-chain of hemoglobins (16). The created uAUG generates a uoORF of 42 nucleotides in the NM_000518.5 transcript of the *HBB* and has been shown to be associated with an increased risk of β-Thalassemia (16). Moreover, Calvo and collaborators demonstrated that the c.-29G>A variant is associated with a decrease of the luciferase activity *in vitro*, suggesting that the presence of the uoORF could alter the levels of the main protein (1).

Disseminated bronchiectasis (DB) is characterized by abnormal dilation of bronchi associated with pulmonary dysfunction. A uAUG-creating variant in the 5′UTR of the *CFTR* gene (NM_000492.3:c.-34C>T) at the origin of a 108 nucleotide overlapping upORF has been described as associated with DB (19). This variant leads to a decrease of the luciferase activity in two different cell lines, in the context of two CFTR isoforms starting at positions c.-132 or c.-69. Moreover, the authors performed additional experiments *in vitro* confirming the recognition of the created uAUG by the ribosomes at the origin of a normal luciferase activity when the uAUG and its Kozak sequence were cloned in frame with the luciferase. These observations strongly support a role of the c.-34C>T variant on the reduction of the translation efficiency at the main ORF by the presence of the uoORF.

The Endoglin (*ENG*) gene is one of the main disease-causing genes for hereditary hemorrhagic telangiectasia (HHT), also known as Osler–Weber–Rendu syndrome, a rare vascular disorder causing abnormal vessel formation. *ENG* can be considered as a special gene with respect to upORFs. Indeed, four rare 5′UTR variants have been described so far in HHT patients to create uAUGs potentially at the origin of upORFs (18, 34–37, 77). These variants are NM_001114753.3: c.-142A>T, c.-127C>T, c.-10C>T, and c.-9G>A. Functional studies have been conducted for three of them (c.-142A>T, c.-127C>T, and c.-9G>A), bringing out an effect of the analyzed variants on the protein levels *in vitro* (18, 35–37, 77). Interestingly, a moderate decrease (∼20%) of the protein levels has been associated with c.-9G>A variant compared to a drastic reduction observed for c.-142A>T and c.-127C>T (∼60% and ∼75%, respectively). These studies also indicate that c.-142A>T and c.-127C>T variants are associated with severe phenotypes while patients carrying the c.-9G>A variant exhibited moderate HHT phenotype. At the molecular level, c.-142A>T, c.-127C>T, and c.-10C>T are predicted to be at the origin of uoORF (270, 255, and 138 nucleotides, respectively). The only exception holds for the c.-9G>A variant, that creates a uAUG in frame with the CDS, and generates an elongated CDS, probably at the origin of a longer form of the ENG protein carrying three additional amino acids. These molecular findings are in perfect concordance with clinical and familial data, suggesting that uoORF-creating variants in *ENG* are causative of a severe form of HHT. Among these four *ENG* variants, c.-9G>A, c.-10C>T, and c.-127C>T but not c.-142A>T are reported in public databases (ClinVar and HGMD). Interestingly, c.-10C>T and c.-127C>T are classified as likely pathogenic in ClinVar but the classification of the c.-9G>A is still conflicting. An additional uAUG-creating variant in the 5′UTR of *ENG* (c.-79C>T) at the origin of a 207 nucleotide uoORF. Of note, even though the c.-10C>T and c.-79C>T variants have not been evaluated in functional studies, one could speculate that in a similar way as for the c.-142A>T and to c.-127C>T variants, these variants would be associated with a reduction of the protein level.

Our group recently identified a disease-causing mutation in the 5′UTR of *PROS1* in an extended family affected with protein S deficiency (PSD) and familial thrombophilia (27). The identified variant was a never reported C>T substitution at c.-39 position creating a uAUG at the origin of an overlapping ORF of 156 nucleotides (NM_000313.4). Using *in vitro* assays, we demonstrated that this variant is associated with a total abolition of protein S levels. With the aim of restoring the main open reading frame in presence of the identified variant, we deleted one base pair at the new stop codon associated to the generated uoORF and, based on the detected protein weight by western blot, identified a protein probably starting at the c.-39C>T-created uAUG. This result indicated that the created uAUG could be used for translation and thus reduces or completely abolishes the translation rate at the main AUG, which explains null protein S level *in vitro* in presence of the variant.

Finally, three additional genes coding for proteins involved in CVDs have been highlighted in Whiffin et al. (15) from public databases as harboring rare uAUG-creating variants.

One is the *F8* gene coding for the coagulation factor VIII, a known susceptibility gene for venous thrombosis (38). The reported uAUG creating variant is the NM_000132.4:c.-5A>G variant that creates an overlapping upORF of 63 nucleotides (20). Very interestingly, this variant is simultaneously predicted to modify a TAA stop codon into a TGA, in frame with two different non-canonical TIS (CTG) generating fully upstream upORFs of 39 and 123 nucleotides. upORFs ending with TGA have been shown to be associated with less translation efficiency of the main protein comparing to TAA ending ones (5). This variant was identified in a patient with mild FVIII activity, an observation compatible with an inhibitory effect on F8 expression of a variant associated with many upORFs. However, even if this variant is reported in HGMD database, its pathogenicity still needs to be validated.

The second gene is *HAMP*, coding for hepicidin whose increased plasma levels have recently been reported to associate with the risk of venous thrombosis (39). Whiffin et al., reported one rare variant in the 5′UTR of HAMP at the origin of a uAUG and catalogued in HGMD. While the *HAMP* variant has been described at the origin of an out of frame uoORF in (15) and described by Matthes and collaborators (17) as potentially generating an abnormal protein responsible for juvenile hereditary hemochromatosis, we did not find any stop codon in the transcript NM_021175.4 sequence that could be in frame with this created uAUG. Thus, this uAUG is unlikely at the origin of an ORF. Nonetheless, one cannot exclude a potential competition between the uAUG and the main TIS regarding the affinity of ribosomes. Indeed, no hepcidin was found in the urine of homozygous patient, suggesting that this variant could alter the translation of the main protein. As for *F8* c.-5A>G, experimental validation of its possible function impact on the translation of the associated protein is still needed.

The last cited gene is *LDLR* implicated in familial hypercholesterolemia associated with increased risk of cardiovascular diseases (40). The deletion of the cytosine at position c.-22 in the 5′UTR of the latest version of the *LDLR* transcript (NM_000527.5) has been identified in a homozygous form in an 8-year-old child diagnosed with familial hypercholesterolemia (22, 41). Interestingly, the c.-22delC is at the origin of an AUG generating an overlapping upORF of 174 nucleotides. This predicted effect could explain the potential pathogenicity of this variant and its association with familial hypercholesterolemia. Nonetheless, the impact of this variant on the LDLR levels still need to be evaluated.

### Common upSNVs Associated With Cardiovascular Disorders and Their Quantitative Risk Factors

In this section, we report the few examples where common upSNVs were identified to be in LD with lead GWAS SNVs (Table 2).

**TABLE 2:** Common upSNVs in GWAS loci for CVDs and associated traits.

upSNV	Gene (orientation)	cDNA position	Genomic position (GRCh38.p13)	Predicted functional effect	GWAS lead SNPs	r^2^/D’ *	References
rs1801020	*F12* (−1)	NM_000505.4 c.-4C>T	chr5:177409531	ACG>ATG uoORF = 9 nts uSTOP = TGA	rs1801020	1.0/1	1
rs492571	*FRMD5* (−1)	NM_001286491.2 c.-487A>G	chr15:43919075	ATA>ATG uORF = 39 nts uSTOP = TAA	rs492571	1.0/1	52
rs75699653	*PEAR1* (1)	NM_001353683.2 c.-491C>T	chr1:156902203	ACG>ATG uORF = 63 nts uSTOP = TGA	rs12566888	(0.00/−1	53
rs58852338	SLC18A1 (−1)	NM_001135691.3 c.-276G (A	chr8:20181901	GTG>ATG uORF (36 nts uSTOP (TAG	rs55682243	(0.00/−1	55
*rs2231861*	*FGF21* (1)	NM_019113.4 c.-173C>G	chr19:48756064	ATC>ATG uORF = 36 nts uSTOP = TGA	rs838133	0.04/−1	56
*rs3811050*	*IL1F10* (1)	NM_032556.6 c.-143C>T	chr2:113072596	ACG>ATG eCDS = 603 nts uSTOP (TAG	rs6761276	0.06/0.74	30
rs35137994	ANGPTL4 (1)	NM_139314.3 c.-140C (T	chr19:8364182	ACG>ATG eCDS = 1362 nts uSTOP = TAG	rs116843064	∼0.00/−1	56, 59, 60
rs3131003 rs3815087	*PSORS1C1* (1)	NM_014068.3 c.-199G>A c.-94G>A	chr6:31125705 chr6:31125810	GTG>ATG uORF = 183 and 78 nts same uSTOP = TGA	rs3094205	0.61/0.97 0.03/0.26	52, 63

*uoORF, upstream overlapping open reading frame, uORF, fully upstream open reading frame; nts, nucleotides; uStop, upstream stop codon, eCDS, elongated coding sequence.*

*Pairwise linkage disequilibrium metrics (r^2^, D’) between upSNV and lead GWAS SNP.

#### F12 rs1801020 (NM_000505.4:c.-4C>T) and Venous Thrombosis

This variant is one of the most well-known and studied common upSNVs. It generates a very small overlapping ORF (nine nucleotides) and has been demonstrated in several independent studies to associate with decreased plasma levels of the clotting factor FXII (42–47). Calvo and colleagues have also demonstrated that this polymorphism is associated with a decrease of the protein levels *in vitro* (1) and that this decrease was due to the creation of the uoORF. While this variant has also been found (48, 49) associated with activated partial thromboplastin time, a biomarker for venous thrombosis, its impact on thrombosis risk is highly debated (1, 42, 47, 50, 51), especially as it never emerged from large-scale genetic association studies on arterial, cerebral, nor venous thrombosis. However, keeping in mind that the effect of a given upORF could be dependent on the cellular environment [e.g., hypoxia (12) and stress conditions (9)], it cannot be excluded that the rs1801020 could be associated with an increased prothrombotic state under certain environmental conditions that need to be further investigated.

#### FRMD5 rs492571 (NM_001286491.2:c.-487A>G) and Lipids

The rs492571 located in the 5′UTR of the *FRMD5* NM_001286491.2, at c.-487 position, is in nearly complete association (*r*^2^>0.80, *D*’ ∼ 1) with several intronic SNPs reported to be associated with triglycerides and HDL-cholesterol levels (52). The A>G substitution at this position is predicted to create a new start codon and could generate a uORF of 39 nucleotides. These observations suggest that the rs492571 could be a good culprit candidate for the observed associations with lipids.

#### PEAR1 rs75699653 (NM_001353683.2:c.-491C>T) and Platelet Aggregation

*PEAR1* was identified as one of the first GWAS loci for platelet aggregation (53) with the intronic rs12566888 (or any polymorphism in strong LD with it) as lead SNV. *PEAR1* harbors one upSNV, the rs75699653, in complete negative LD (*D*’ = − 1) with rs12566888. Because of the difference in their allele frequencies, the minor allele frequency of the former being ∼0.02, that of rs12566888 being ∼0.09, their pairwise LD *r*^2^ is close to null. However, they generate three haplotypes where the rs75699653-T allele, predicted to be at the origin of a uORF of 63 nucleotides, is always carried by the rs12566888-G allele (Supplementary Table 1). Interestingly, the rs1256688-G allele is either positively or negatively associated with platelet aggregation depending on how platelets are stimulated (54). Haplotype association analysis of these two SNVs in relation with platelet aggregation would be mandatory to determine if the original GWAS signal could be (partially) explained by the rs75699653-T carrying haplotype.

#### SLC18A1 rs58852338 (NM_001135691.3:c.-276G>A) and Triglycerides

*SLC18A1* is one of the numerous loci associated with triglycerides levels (55). It harbors in its 5′UTR one upSNV, rs58852338, whose minor T allele (corresponding to c.-276A on the antisense transcript) with frequency ∼1% is predicted to create a uORF of 36 nucleotides. The rs58852338-T allele is always carried by the haplotype carrying the rs55682243-C allele that was observed to associate with decreased triglycerides levels (55). This case is then similar to the *PEAR1’s* discussed above.

#### Fibroblast Growth Factor 21 (FGF21) rs2231861 (NM_019113.4:c.-173C>G) and Triglycerides

*FGF21* is another locus identified by GWAS as influencing triglycerides levels in plasma (56). The lead SNV is the synonymous rs838133 that does not show strong LD with any other SNVs when one uses the pairwise *r*^2^ threshold of 0.80. However, it is in complete negative LD (*D*’ = − 1) with the rs2231861 upSNV. As a consequence, these two SNVs generate 3 haplotypes. As for the two previously described examples, the rare rs2231861-G allele predicted to create a uORF of 36 nucleotides is always carried by the haplotype harboring the rs838133-G allele associated with decreased triglycerides (56). Of note, the latter has also being found associated with decreased levels of homocysteine (57), another cardiovascular biomarker.

#### IL1F10 rs3811050 (NM_032556.6:c.-143C>T) and Coronary Artery Disease Risk

One common upSNV is present in the 5′UTR region of the *IL1F10* gene, a susceptibility locus for myocardial infarction (MI) (30). This is rs3811050 where the rs3811050*-*T is predicted to create an eCDS of 603 nucleotides while the canonical CDS is of 459 nucleotides. At *IL1F10*, the rs6761276-T allele of the missense p.Ile44Thr was found to be associated with increased risk of MI (30). According to the variant effect predictor (VEP) tool (58), the predicted pathogenicity of rs6761276 could be transcript-dependent^4^. It makes then sense to hypothesize that the impact on MI of the rs6761276-T allele may be different according whether or not it is present on the eCDS. As a consequence of their LD pattern (*D*’ = 0.74, *r*^2^ = 0.06), the rs6761276 and rs3811050 generate 4 haplotypes among which one (frequency ∼0.015) is carrying both the rs6761276-T risk allele and the eCDS rs3811050-T creating allele. It would be interesting to determine whether this specific rare haplotype is more at risk of MI than the haplotype carrying the rs6761276-T risk allele but not the eCDS creating allele.

#### ANGPTL4 rs35137994 (NM_139314.3:c.-140C>T), and Cardiovascular Traits

The *ANGPTL4* gene is an interesting locus for CVD as it has been shown to associate with several cardiovascular phenotypes, including CAD risk (59), lipid-related (56, 60), and red blood cells (61, 62) traits, with lead SNV being the missense rs116843064 (p.Glu40Lys) polymorphism. The minor rs116843064-A allele, with frequency ∼1% is associated with decreases in CAD risk, in triglycerides levels, in reticulocyte counts and with increases in HDL levels, mean corpuscular volume and red cell distribution width. *ANGPTL4* harbors in its 5′UTR a common upSNV, rs35137994, whose minor T allele with frequency ∼5% and that is predicted to generate an eCDS of 1362 nucleotides. However, due to complete negative LD (*D*’ = −1), the rs116843064-A and rs35137994-T alleles are never present on the same haplotype, indicating that the effect of the rs116843064-A allele cannot depend on whether it is present on the elongated isoform. As a consequence, the upSNV is unlikely to explain the observed GWAS signals, especially as the missense rs116843064 is predicted to be deleterious according to several standard prediction tools such as PolyPhen (probably damaging), SIFT (damaging), and CADD (score 31.0). That said, given these current observations, one cannot completely exclude that the rs35137994-T could exert additional independent and less pronounced effects on the aforementioned CVD traits.

#### PSORS1C1 (NM_014068.3: c.-199G>A and c.-94G>A) and Lipids

The last discussed GWAS locus is *PSORS1C1* that has been associated with plasma triglycerides levels (52) and hemoglobin levels (63). This locus has also been found associated with Psoriasis (64). *PSORS1C1* presents with two common upSNVs in its 5′UTR region, rs3131003 (c.-199G>A) and rs3815087 (c.-94G>A) with minor allele frequencies of ∼0.40 and ∼0.20, respectively. Individually, these two upSNVs could be at the origin of two uORFs of 183 and 78 nucleotides, respectively, both terminating at the same stop codon at c-19. However, because of complete positive LD (*D*’ = 1), the rs3815087-A is always associated with the rs3131003-A allele, meaning that the predicted uORFs always exist together, with the 78 nucleotide length uORF always included in the longer one of 183 nucleotides. Whether this could result in one or two small peptides depends on the competition between the created uAUGs and remains to be elucidated. The rs3131003 is also in nearly complete positive LD (*D*’ = ∼ 0.97, *r*^2^ ∼ 0.60) with the rs3094205 lead SNV associated with triglycerides, suggesting that the former could be a good candidate for explaining the GWAS signal.

Of note, we did not observe any common upSNVs that exhibit strong LD with stroke- nor VT-associated lead SNVs and that could then explain the GWAS signals observed at their locus.

## Discussion/Perspectives

While there is increasingly awareness of the impact of rare upSNVs in rare Mendelian disorders, there has been so far little initiative to investigate the possible role of such variants in the susceptibility to common diseases and their quantitative risk factors. From a list of ∼700 loci identified in GWAS for CVD traits, we only identified a very minor proportion of loci (5: *FGF21, FRDM5, PEAR1, PSORS1C1*, and *SLC18A1*) where the GWAS signal could be partially explained by upSNVs. We focused here on CVDs but similar investigations merit to be conducted for other human diseases. Our results were based on *in silico* observations (bioinformatics predictions coupled to LD analyses) and deserve to be further investigated through fine-mapping association analysis and experimental molecular characterization. Several molecular techniques (gene reporter assays, toeprinting, polysome profiling, among others) are available to evaluate the effect of upSNVs on the translation machinery and/or protein expression. Here, we would like to highlight the recent advances in the antisense oligonucleotides (ASOs) strategy targeting upORFs, as it also offers therapeutic perspectives in the context of rare diseases. ASOs are very efficient molecular tools designed to modulate gene expression through Watson–Crick base pairing with specific motifs on target transcripts (65, 66). Initially, ASOs were used to downregulate gene expression or to modify RNA splicing. Recently, ASOs have been proposed to ameliorate gene expression by directly targeting uAUG (67). Liang et al., have shown that this technique depends on many factors on the RNA and on the chemical structure of the used ASOs (67). However, targeting upORF using ASOs seems to be a very innovative and efficient genetic tool to assess *in vitro* the functional impact of upSNVs on protein levels. Beyond their *in vitro* utility, effective ASOs capable of restoring protein levels could be used as a therapeutic approach to treat rare diseases caused by upSNVs. ASOs have indeed demonstrated great potential for treating rare diseases (68–71) due to coding or splice mutations. The antisense field has remarkably progressed over the last few years with the approval of several antisense drugs and with the development of even more potent compounds (72), opening promising perspectives to treat upORF-altering variants.

In this analytic review, we focused on SNVs known, or predicted, to create upORFs. We did not discuss molecular tools that are available to determine whether these upORFs could be at the origin of functional small micropeptides that could have specific physiological roles. This topic has recently been addressed in an independent review (28). Finally, we only examined in this work SNVs that could create uAUG resulting upORFs, the most known class of variants among those that affect non-canonical ORFs. Ribosome profiling data have shown the presence of small ORFs (sORFs) in coding transcripts outside the 5′UTR but also in non-coding RNAs (73, 74). Some of these sORFs have been shown to be translated into small encoded peptides and/or to have a regulatory role on gene expression (75, 76). Thus, one can easily speculate that genetic alterations in such sORFs could also have functional consequences and be involved in human diseases. The next steps would then be to characterize the spectrum of SNVs creating or deleting TIS or Stop in non-coding transcripts.

## Author Contributions

CM and DA developed and applied the MORFEE bioinformatics tool. OS and D-AT designed the study, conducted the systematic review, and drafted the manuscript. OS and CP performed *in silico* annotations of the predicted upORFs. AG and ME completed the manuscript. All authors contributed to the article and approved the submitted version.

## Conflict of Interest

The authors declare that the research was conducted in the absence of any commercial or financial relationships that could be construed as a potential conflict of interest.

## Publisher’s Note

All claims expressed in this article are solely those of the authors and do not necessarily represent those of their affiliated organizations, or those of the publisher, the editors and the reviewers. Any product that may be evaluated in this article, or claim that may be made by its manufacturer, is not guaranteed or endorsed by the publisher.

## Footnotes

1
https://github.com/daissi/MORFEE

2
https://ldlink.nci.nih.gov/?tab=home

3
https://grasp.nhlbi.nih.gov/Overview.aspx

4
https://gnomad.broadinstitute.org/variant/2-113832312-T-C?dataset=gnomad_r2_1
